# Active listening, shared decision-making and participation in care among older women and primary care nurses: a critical discourse analysis approach from a gender perspective

**DOI:** 10.1186/s12912-024-02086-6

**Published:** 2024-06-17

**Authors:** Pablo Martínez-Angulo, Manuel Rich-Ruiz, M. Rocío Jiménez-Mérida, Salvador López-Quero

**Affiliations:** 1 Department of Nursing, Pharmacology, and Physiotherapy, Faculty of Medicine and Nursing, University of Córdoba (UCO), Edificio Servicios Múltiples. 1St Floor. Avda. Menéndez Pidal S/N, 14004 Córdoba, Andalucía Spain; 2 Interdisciplinary Research Group in Discourse Analysis (HUM380), University of Córdoba (UCO), 14071 Córdoba, Spain; 3 Instituto Maimónides de Investigación Biomédica de Córdoba (IMIBIC), Hospital Universitario Reina Sofía (HURS), 14004 Córdoba, Spain; 4 Ciber Fragility and Healthy Aging (CIBERFES), Nursing and Healthcare Research Unit (Investén-Isciii), Instituto de Salud Carlos III, 28029 Madrid, Spain; 5 Faculty of Philosophy and Letters, University of Córdoba (UCO), 14003 Córdoba, Spain

**Keywords:** Critical discourse analysis, Gender, Older women, Primary care nurses, Active listening, Shared decision-making, Active participation, Power relations

## Abstract

**Background:**

Nursing care for older women represent a challenge worldwide due to its characteristics. When communication is impaired between primary care nurses and older women living alone, an imbalance in power relations occurs. The main objective of this study is to analyse the power relations between older women and primary care nurses in situations of active listening, shared decision-making and participation in care.

**Methods:**

We developed a qualitative study in southern Spain using a discursive and gender approach. We used purposeful sampling to interview older women who lived alone and received home nursing care. Simultaneously, we conducted focus groups with primary care nurses who provided home care to older women. A linguistic analysis of the transcripts was carried out.

**Results:**

Nine semi-structured face-to-face interviews were conducted with older women who lived alone and two face-to-face focus groups with four primary care nurses in each. The discourse of the participants demonstrated an imbalance in power relations. Influenced by work overload, active listening was considered a privilege in primary care nurses´ discourse. Regarding shared decision-making, older women´s discourses revealed “mirages” of real situations where they thought they were deciding. Participation in care was difficult since older women saw themselves as a nuisance in nurses´ presence, and primary care nurses did not facilitate older women’s engagement. Older women weren´t considered when organising home visits and had interiorised a subordinated feeling. Similarly, a strict sense of identity made primary care nurses feel powerful in their relationships with older women.

**Conclusions:**

The discourse of older women represented them as victims of a hostile panorama whilst they were sometimes satisfied with the deficient care received. The discourse of primary care nurses used more discursive strategies to represent themselves as professionals committed to caring. However, it also revealed deficiencies in care, discriminatory elements, and feelings of being limited by their working conditions. Active listening to older women and engagement in decision-making readjust empower the older women. Attending to the needs and concerns of primary care nurses could recalibrate the power imbalance between them and healthcare organisations.

**Supplementary Information:**

The online version contains supplementary material available at 10.1186/s12912-024-02086-6.

## Introduction

Nursing care in the older population presents a particularly complex challenge due, among other factors, to multiple pathologies, high morbidity, and frailty that characterise this profile of patients [1]. This complexity is not always reflected in the moments of care nor in how the nurse-older-patient relationship is established, creating an image of power imbalance in nursing care. The imbalance is also accentuated by issues related to sex and gender [2, 3]. Several studies have shown that older women have a longer life expectancy, reaching an advanced age with more significant functional impairment [4, 5]. On the other hand, traditional gender regulations —to which women continue to be linked in an industrialised society— are replicated in the current social framework and the health systems, favouring health inequities and limiting access to proper care [6]. For this reason, the nursing personnel and research within the nursing discipline must consider these aspects framed in care.

To achieve this purpose, the power relations between nursing professionals and older women in the community setting should be addressed, focusing on detecting possible power imbalance and inequality among them. Within sociocritical studies, the feminist perspective provides models to elucidate the dimensions of power and the influence that history, culture, and society exert on this interaction [7]. It is therefore pertinent to explore the perspective of women involved in community nursing care processes from the angle of empowerment and equity in health, breaking down stereotypes such as ageism and social prejudices, to progressively engage older women in decision-making moments of their care process [8].

From a discursive approach, nursing care is based on establishing an efficient, communicative relationship with patients and their environment. This relationship should be bidirectional and promote, above all, quality care [9]. However, this relationship does not always correspond to reality, and therapeutic communication can be altered. Proper care will only be possible by listening and, thus, knowing older women's needs, preferences and personal values concerning the health-disease process framed in their sociocultural context [10]. To achieve this, it is essential to identify power imbalance in listening to preferences, making decisions and participating in care, and contribute to creating an equal world in which the dignity and rights of older people are respected, a group prone to being vulnerable, especially in the case of women [11, 12].

In pursuit of this, previous studies have addressed the nurse-patient relationship in the older population. Mize et al. [13] focused their research on older women regarding interaction and nurse relationships. On the other hand, Nilson et al. [14] studied how a patient-centred approach influenced improving healthcare for older patients. This perspective empathised with a holistic and systematic approach to nursing care, not limited to a specific moment but being longitudinal concerning effective and fluid communication between the nurse-patient and nurse-institution pairings.

However, patient-centred communication is not necessarily linked to shared decision-making (SDM) [15]. We understood SDM as the communicative encounter in which patients and health personnel make a joint decision that best suits the situation, thoughts, and patient preferences [16, 17]. Likewise, while introducing SDM, patient preferences and active participation in care, we should speak of patient-centred care as a whole that interconnects these three elements through patient-centred communication [18, 19]. Thus, in the case of older people, this patient-centred care is at greater risk of being ignored as, on many occasions, health personnel shape older patients' opportunities to actively participate depending on their functional qualities [20]. In this regard, the present study contemplates patient participation as an active patient contribution throughout the care process. This contribution can be made by asking questions, expressing concerns, or expressing preferences [21]. Active patient participation is also part of patient-centred care and requires SDM [22]. In this sense, active listening (AL) is the effective use of communicative tools that establish a communication relationship that boosts patient participation [23], representing a powerful device to appease negative feelings such as loneliness that can stigmatise older people themselves [24]. In those cases, ineffective communication prevents detecting these feelings and situations [24, 25]. For this reason, in situations where poor communication characterises the interactions between health personnel and older people, SDM and participation in care are affected by signs of mistrust, exclusion or discrimination in care [26–28].

Since the interaction between older people and nurses has proven to be crucial [29], the principal aim of this study was to critically analyse the power relations between older women and primary care nurses. Subsequently, the specific objectives were to describe the situations of AL to preferences, SDM, and patient participation from older women and primary care nurses´ discourse; to identify differences in the perception of those situations between older women and primary care nurses´ discourse; to explore the discursive strategies that shape the social representations projected by older women and primary care nurses´ discourse.

## Methods

### Study design

We performed a qualitative study framed in a critical discourse analysis (CDA) approach with a gender perspective. CDA is not considered a single methodology but a set of methodological frameworks centred around an analysis linking society and discourse's linguistic component [30, 31]. This study uses Van Dijk's Power Relations Theory as a reference considering his sociocognitive approach [32, 33] and Foucauldian elements such as oppression and society [34]. This model focuses on the discursive sources of power, dominance, inequality and bias**,** connecting with Bourdieu's Social Practice Theory (SPT) [35, 36]. SPT frames the relationship between gender and symbolic power in a social web where health behaviours in social groups can change due to inequality. Thus, discourse, in addition to power, reproduces gender as a social identity construct [37]. The CDA, combined with a gender perspective, attends to the social representations of gender —identities— and the role of gender in power relations within specific contexts [38, 39].

To report our qualitative study, in Additional file 1, we present the completed SRQR checklist with the page and paragraph numbers stated for each item [40].

In addition, to offer a detailed and complete report of the interviews and focus groups, Additional file 2 contains the completed COREQ checklist for original qualitative studies [41].

Likewise, for an adequate presentation of information on sex and gender throughout the present study, the considerations reflected in the SAGER guidelines have been followed [42].

### Setting and sample

The study was conducted in the Córdoba-Guadalquivir Health Area community care setting, specifically in two centres. The province of Córdoba is located in the north centre of the Autonomous Community of Andalusia (Southern Spain). According to data corresponding to the year 2021 extracted from the Institute of Statistics and Cartography of Andalusia, the province’s total population is 322,071 (167,454 women and 154,617 men), whose rate of the population over 65 years is 19.6%. Within this community care setting are thirteen primary care centres and nine clinics in rural areas. One of the two health centres was responsible for the primary health care services of the sixth poorest suburban district in the country, according to the Urban Indicators in its 2022 edition of the National Institute of Statistics of Spain [43]. Concerning nursing care in this study, it is offered universal healthcare and paid for by taxpayers through social security. The older patients who received nursing care at home were previously assessed by nurse case managers who, based on the needs raised by the patients, decided to enhance the home care services that were carried out by a team of health professionals with bachelor’s degrees in nursing belonging to their respective health centres and had home care among their tasks.

We performed purposeful sampling according to the selection criteria [44]. These criteria are included in Table 1.

**Table 1:** Selection criteria for older women and primary care nurses

	**Older women**	**Primary care nurses**
Inclusion criteria	A. 75 years old or older B. Living alone at home C. Receiving nursing home care services at the time of the study	F. Having at least two months of work experience in primary care settings G. Having at least two months of uninterrupted work experience as a primary care nurse in the health centres of the study H. Having nursing home visits to older women as part of the primary care nursing tasks
Exclusion criteria	D. Scoring a suspicion of cognitive impairment in the Spanish version of the Pfeiffer test E. Suffering from a terminal illness	I. Not having made at least one nursing home visit per month to older women in the last two months since the study

Regarding recruiting older women, the leading researcher transferred the selection criteria for this group to the nurse case managers in the health centres. The nurse case managers contacted potential participants through nurses who worked in each health centre. A three-way meeting was held between all interested older women individually, a primary care nurse, and the leading researcher. After explaining all the study information, the leading researcher arranged a second meeting with each older woman to conduct the interview.

The corresponding selection criteria were told to the nurse care managers about recruiting primary care nurses. These criteria were sent to nurses working in the health centres, and those interested in participating contacted the leading researcher.

### Data collection

On the one hand, we decided to conduct semi-structured interviews with older women because this tool facilitates some critical points to pivot the data collection and not limit the depth of the life experience of older women [45, 46]; on the other hand, we used the focus group in the case of the primary care nurses for the following reasons: (1) achieving greater dynamism in the sessions, (2) finding a sense of the experience lived as a socio-professional group, as well as intra-group norms, codes and ideologies, (3) allowing the discourse to act as a vehicle for revealing discursive strategies in the natural communicative habitat between member group [32, 33], (4) triangulating qualitative methods [45].

According to the wishes of the older women, the interview spot was their own home; an indoor and conditioned enclosure in their respective health centres, concerning primary care nurses. All the interviews and focus groups were audio-recorded and accompanied by a reflexive diary that comprised self-hermeneutical considerations, kinesics and proxemics [47]. Interviews were anonymised by randomly giving each participant a number, and the leading researcher reviewed the transcripts to ensure accuracy. Semi-structured interviews were approximately 60 min on average, and focus groups were 50 and 70 min. The formal caregivers of patients 4, 6 and 7 were also present during the interviews. Likewise, two observers from the research team were present separately and took field notes in each focus group. The leading researcher was the interviewer in the case of the older women and the group moderator in the case of the primary care nurses.

We obtained older women´s health information about their medical and nursing records through the nurse case managers. As for the exclusion criterion D for older women, the reference cut-off point was four or more errors since all the older women participating in this study had great difficulties reading and writing [48]. On the other hand, the socioeconomic level was obtained by self-report through a direct question to each participant outside the interview script provided.

Regarding data collection instruments, we developed a preliminary script which was tested with two older women who met the same selection criteria. These primitive interviews were not incorporated into the final corpus of the study because we did this to calibrate its content a posteriori and assess whether we focused correctly on the interview encounter. Once the final script was approved, interviews were conducted with the older women selected based on the abovementioned criteria. Regarding primary care nurses, we carried out the focus groups once the interviews with the older women were done, adapting the script to have the same skeleton of content and to address the groups. Nevertheless, this script was used as a reference as the dynamic nature of focus groups determined the course of the sessions (see Additional files 3a & 3b).

### Data analysis

We employed a linguistic analysis defined by CDA that addressed the communicative event regarding the study phenomena [49, 50]. Through the linguistic analysis, we aimed to explore the discursive strategies that unravel the positive representation of the *self* (semantic macro-strategy of in-group favouritism) and the negative representation of the *other* (semantic macro-strategy of derogation of the out-group). We identified these discursive strategies representations by analysing speech acts that illustrate authority figures, comparisons, exemplifications, generalisations, polarisations, presuppositions, and victimisations [51, 52].

For the determination of the research corpus, we followed the steps recommended by Bolívar [53]: (a) to differentiate the textual corpus from the research corpus, (b) to select informative material through an awareness of critical assumptions and problems to be addressed, (c) to determine the linguistic sublevels to approach.

After obtaining the research corpus, we chose to approach the analysis of the pragmatic, syntactic, semantic, rhetorical-stylistic and cognitive sublevels, as well as a description of the discursive strategies deployed by the participants.

### Trustworthiness and rigour

We assessed the trustworthiness and rigour of our study through the rigour and quality criteria suggested by Lincoln and Guba [54]. In addition to a detailed explanation by the team, we also identified the rigour techniques we used to address each criterion [55, 56]. This information can be seen in Table 2.

**Table 2:** Steps taken to ensure trustworthiness and rigour. Adapted from Lincoln & Guba [54]

Lincoln & Guba’s trustworthiness criterion	Techniques used for ensuring study quality, according to Lincoln & Guba	Researcher response
1. Credibility	1.1. Member checking	During and at the end of the interviews and focus groups, the interviewers repeated and summarised the participants´ answers to ask for clarification and confirmation of the researcher’s interpretation of the answers. Moreover, the interviewer also asked the participants a final question about possible comments they wished to make regarding what had been said and potential topics not raised through the interviews but that they wanted to make explicit
	1.2. Referential adequacy	The research team involved in the data collection and analytical phase maintained a constant dialogue between the analytical results and the raw data obtained. They carried out this continuous back-and-forth path to achieve appropriate adequacy and fidelity to the participants' discourses
	1.3. Triangulation	In this study, we used data collection tools such as semi-structured individual interviews and focus groups to ensure a triangulation of techniques. Likewise, in each focus group, a different research team member played the observer role, collecting field notes so that the perceptions of several researchers could be compared. We have also ensured our results through researcher triangulation, sharing and discussing decisions and findings. The last researcher in this study, an expert in CDA, supervised the analysis process in parallel, raising the results reciprocally and agreeing with the final results
2. Transferability	2.1. Thick description	To enable a comparison of the context of this study with others and allow its transferability, we have compiled a thick description of the characteristics of the participants, collected in Tables 2 and 3. At the same time, we have explained the setting as comprehensively as possible to facilitate a transfer of the context
3. Dependability	3.1. Use of overlap methods	[See response to 1.3.]
	3.2. Inquiry audit	We handed over the project of this study to an expert researcher in qualitative research outside the research team of this work, who reviewed the different phases and how we conceived them. In addition, we discussed with the expert the moments of decision that would be carried out during the study
4. Confirmability	4.1. Triangulation	[See response to 1.3.]
	4.2. Reflexivity	The research team was aware of its preconceptions about the study phenomenon. To safeguard the richness of meanings generated by intersubjectivity and avoid biases, the leading researcher carried out a process of self-hermeneutics that he periodically reflected on in a reflexive diary. The leading researcher, using a theoretical framework of inequality in power relations as a reference and a gender perspective, was aware that his personal values and commitment matched these theoretical precepts, which gave even more strength to his inquiry attitude

### Ethical considerations

This study received approval from the Research Ethics Committee of Córdoba. We informed all the participants about the research before the data collection through an information sheet and informed consent to participate was collected by signing. We explained to the participants that the data collected would be used for research purposes only and that all identifying information would be anonymised to safeguard their identity. We informed the participants about (a) the objectives of the research, (b) the guarantee of the confidential nature of personal data, (c) the custody and handling of the data, (d) the disclosure of the results of this research, (e) the possibility of leaving the study at any time and without any consequences.

## Results

### Description of the participants

In the case of older women, we conducted nine face-to-face semi-structured interviews, which were determined by the discourse saturation criterion agreed upon between the research group in a discussion meeting [54]. In the case of primary care nurses, we performed two focus groups of four nurses each.

Older people were all Spanish white women, with a mean age of 87 (Table 3).

**Table 3:** Characteristics of older women (*n* = 9)

Participant No	Age	Medical diagnosis	NANDA nursing diagnosis	Pfeiffer test score Spanish version in no. of mistakes	Barthel index score out of 100 (meaning)	Norton scale score out of 20 (meaning)	Social support (type)	Economic difficulties (with help)
1	95	Bladder cancer (operated), cataracts	Activity intolerance, impaired urinary elimination, risk for falls	0	95 (slight dependency)	17 (without risk)	Yes (family)	No (no)
2	88	Hip replacement, hypertension, Ménière's disease, osteoarthritis	Anxiety, bathing self-care deficit, constipation, dressing self-care deficit, impaired home maintenance, impaired physical mobility, impaired walking, risk for falls, risk for loneliness, risk for social isolation	3	85 (moderate dependency)	14 (at risk)	No	No (no)
3	78	COPD, smoking	Activity intolerance, impaired gas exchange, ineffective breathing pattern, risk for impaired skin integrity, risk for falls	0	80 (moderate dependency)	14 (at risk)	Yes (friends)	No (no)
4	84	Obesity, type II Diabetes Mellitus	Activity intolerance, imbalanced nutrition, ineffective health management, sedentary lifestyle	2	85 (moderate dependency)	16 (without risk)	Yes (formal caregiver)	Yes (yes)
5	97	Duodenitis, gastritis, hypertension, mild renal failure, mitral regurgitation	Activity intolerance, imbalanced nutrition, impaired urinary elimination, ineffective health management, risk for impaired skin integrity, risk for falls	3	25 (severe dependency)	10 (at risk)	Yes (family)	No (no)
6	86	Atrial fibrillation, epicondylitis, hypercholesterolemia, hypertension	Activity intolerance, chronic pain, hearing impairment, impaired physical mobility, risk for falls	1	90 (moderate dependency)	17 (without risk)	Yes (informal caregiver)	No (no)
7	90	Colonic diverticulitis, coxarthrosis, discarthrosis, dizziness, glaucoma, gonarthrosis, hypertension, ischemic heart disease, osteoporosis, type II Diabetes Mellitus	Functional urinary incontinence, impaired physical mobility, impaired home maintenance, ineffective coping, risk for falls	3	55 (severe dependency)	12 (at risk)	Yes (formal caregiver)	No (no)
8	84	Atrial fibrillation, breast cancer, colon adenocarcinoma, knee osteoarthritis, obesity	Chronic pain, functional urinary incontinence, imbalanced nutrition, ineffective health management, risk for falls	0	90 (moderate dependency)	17 (without risk)	Yes (family)	No (no)
9	83	Osteoporosis	Functional urinary incontinence	2	80 (moderate dependency)	17 (without risk)	Yes (family)	Yes (no)

Primary care nurses were all Spanish white women, with a mean age of 50 years in group X and 57 years in group Y (Table 4). The corresponding sociodemographic information was collected through a self-filled registration sheet before each focus group.

**Table 4:** Characteristics of primary care nurses (*n* = 8)

Focal group No	Participant No	Age in years	Qualified nursing professional in years	Nursing professional employment in years	Nursing professional employment in the current health centre in years (months)
X	10	42	21	17	2 (6)
X	11	62	42	42	13(0)
X	12	52	32	32	16(0)
X	13	43	23	22	7(0)
Y	14	58	34	34	21(0)
Y	15	63	40	40	13(0)
Y	16	56	24	24	2(0)
Y	17	52	27	27	25(0)

### Conceptual map for the synthesis of the results

**Fig. 1:**
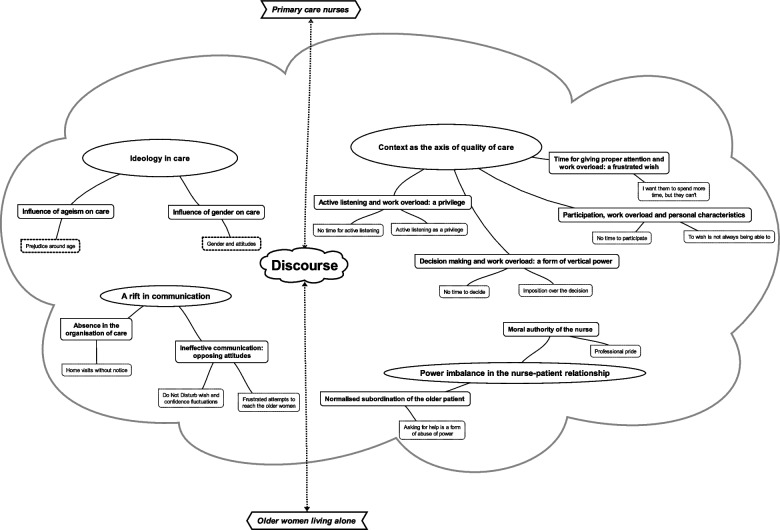
Discursive elements and their power relations between older women and primary care nurses

Visually, Fig. 1 exemplifies how the discourses of primary care nurses and older women shape the reality of AL, SDM, and participation in care.

### Narrative development of the results

The results of this study show explicit representations of power imbalance in the form of speech acts belonging to the discourses. The older women adopted a predominantly subordinate role, while the primary care nurses, despite trying to convey an image of commitment to the care they provided, adopted a dominant role. However, according to the differences in both perceptions, older women and primary care nurses were responsible for ineffective communication, a lack of SDM, and an unrealistic view of participation in care.

To facilitate the comprehension of the results, we present them in Table 5 in an organised manner as major themes, minor themes, and the extracts of speech acts that integrate them.

**Table 5:** Major themes, minor themes and informative excerpts of CDA from the research corpus

**Major theme 1: Influence of ageism on care**	
**Minor theme 1: Prejudice around age**	
**Primary care nurses**	
(1) **I have [she names an older woman], who has already fallen many times, she has a walking frame at home. I tell her to lean on it, to try… and I come another day, and she tells me she got stitches on the head because she fell. And no matter how hard you try, they don't change (no. X/10)** (2) **Older people think their problem is solved with pills (no. X/10)** (3) **It looks like the older patients, you already know that it doesn't matter if you talk to them that you already know that they come to you to prescribe pills and that it doesn't matter what you speak to them that when they leave the door, they will do what they want… (no. X/10)** (4) **She's not old, and you tell her something. She says, “that's because I'm old?”, what if I take the walking frame, am I old? And she is 90 years old, but that is your security, it is her security, so then… (no. X/10)** (5) **[Referencing (4)] But they are not old, “I am not old”, no… (no. X/11)** (6) **Where do you see the supposed inequality… between old and young, where do you see it? (no. X/12)** (7) **It occurs in numerous older people; they have another vision. And you should have it too because that is what I have told you. For instance, they are at the expense of having food brought to them or of being unable to make meals and in the end, the only thing that solves their diet is the tinned food they buy (no. X/13)** (8) **Many young patients with wound care have already seen what is good and what is not. Now you have to be more… tactful, in explaining the procedure (no. Y/17)**	
**Major theme 2: Absence in the organisation of care**	**Major theme 3: Influence of gender on care**
**Minor theme 2: Home visits without notice**	**Minor theme 3: Gender and attitudes**
**Older women**	**Primary care nurses**
(9) **Well… they call me. And I have an appointment with the hairdresser. What do I tell the hairdresser now? That I'm not going? And what do I say to the nurse? Do not come? [she laughs] Yeah, I question, I call into question (no. 1)** (10) **[Interviewer asks referencing (9): and what do you do?] Well, go to the hairdresser's, because I feel fine and don't need the nurse right now… And I need the hairdresser's because I'm going out. And I decide to go to the hairdresser (no. 1)** (11) **No, the nurses come directly, and if I'm not there, well, the little angels leave. I can't demand an hour from them because they have much work (no. 4)** (12) **No, sometimes she comes because she had to; she always had a fixed day, the last two days of the month she always came. And she no longer called; she showed up… Oh, [she names the nurse], how are you coming and not… “Don´t you know already that I come these days?” (no. 6)** (13) **[Referencing (12): and do you tell the nurses what you prefer…?] Well, I´d prefer that she reports me because since I'm alone, sometimes I'm in a housecoat, sometimes I'm… [she laughs] (no. 6)** (14) **Since I am alone here, she comes when she can. And now with more reason since a few of them have gone on holiday… (no. 7)** (15) **No, she usually calls me and tells me, “Look, I'm going to come in”. Well, okay, that´s it, I know she's coming, well… I'm here ready, no worries (no. 8)**	(16) **When a diabetic man comes here, I usually tell him, tell him to come with his wife too, because she is also the one who… (no. X/10)** (17) **[Referencing (16)] Who is the one who cooks the meal for him (no. X/13)** (18) **But maybe I have a more paternalistic attitude than my colleagues, and I would often like to get rid of it, but I don't know. I don't know why I don't know how to say no to people either. Everything they blame me, they trick me, and I say yes. So that leads to much sleep being taken away, but oh well. So there are things that I know I'm not doing well (no. Y/17)**
**Major theme 4: Ineffective communication: opposing attitudes**	
**Minor theme 4: Do Not Disturb wish and confidence fluctuations**	**Minor theme 5: Frustrated attempts to reach the older women**
**Older women**	**Primary care nurses**
(19) **If I feel bad, I count on my sister. That´s it. I have more confidence in her (no. 1)** (20) **I say many times, I think for myself. (…) I think so. I think about it to myself, but not to… tell anyone (…) But, yes, I had to say to the nurse (no. 1)** (21) **If she [the nurse] has to do something else, that's it, and she knows that I'm cared for, that I have a lady, that I'm not alone (no. 2)** (22) **It seems… I don't care. But since I have no problems… What do I say? [she laughs] (no. 2)** (23) **I tell her [the nurse] often that the afternoons seem very long for me (no. 2)** (24) **It makes me very tired of that. It is because I say the nurses have their work, they have their check-ups, their things and I am not going to spoil them (no. 3)** (25) **I don't want to bother my children either. And when something hurts me half the time, I don't even tell them. (…) Because I don't want them to suffer! I'm suffering a lot… (no. 3)** (26) **Never. No, no, it's just that I've never told the nurse. If she changes the visit or whatever, she'll know why. “Don't you have it… like this…?” With love [clarifying in what tone the nurse says the previous]. “Haven't I told you I go on the last two days of the month? On the 24th and 25th.” And I don't… I have no complaint that she doesn't tell me or… I don't even try to say to her or ask her. I let her do it. Because she is worth a lot. Much. I don't ask her anything; she is doing… Going up, going down the day she can, and the day she can't, she doesn't come, and nothing happens (no. 6)** (27) **I let her do it, come on as if she were my mother (no. 6)** (28) **It seems that… refusing her job when she takes it like this… If she takes those pills to me, I need them. And when I don't need them, she tells me to leave them there [the older woman points out a box] (no. 6)**	(29) **Many say, “their nurse had to tell the patient to wash”. And you say… “let's see. Do I tell him…? How do I tell him?” Well… Sometimes… and if you tell him, he gets offended. And the wife asks for another nurse because she says I made her see that she did not take good care of her husband (no. X/11)** (30) **You go to their house, and that´s tough already. “Where does that carpet come from?” They tell you the story of that carpet. That she brought it from… “Excuse me? You´re going to take it away? Why? This furniture… what? My husband and I put it up, this piece of furniture here isn´t going anywhere…”. But look, the walking frame does not fit. Well, I'm left without a walking frame! But I don't remove that piece of furniture or the carpet [she laughs] (no. X/11)** (31) **The problem is with them [the older women]; of course, you intervene! Another thing is… what they want… (no. X/11)** (32) **A woman, one hundred years old, living alone, with her perfect head. She manages her things, goes to the field on the arm of the caregiver, and has a caregiver in the morning and another at night. And her legs were the size of; I don't know… like a column. And you say, “What about the diuretics?” “I take half”… Come on… (no. X/13)**
**Major theme 5: Active listening and work overload: a privilege**	
**Minor theme 6: No time for active listening**	**Minor theme 7: Active listening as a privilege**
**Older women**	**Primary care nurses**
(33) **Neither one thing nor the other. Sometimes they listen, and other times well… Or they think they see me better, well, and… (no. 1)** (34) **She says she also has to attend to many people. She says, “It's just not you alone, but… it's another, another, another. One day I dedicate to one, another day I dedicate to another” (no. 2)** (35) **Of course, I don't think she [the nurse] won't listen to me. That everyone listens, right? Although later they say, look, this is how it is, it is like that, and it's over (no. 5)** (36) **Even if she doesn’t want to, she has to listen to me, whatever I tell her, right? And she comes running, and maybe she can stop; if I talk to her about anything, the woman should listen to me (no. 5)**	(37) **It´s not always possible because there are many, and they want you to be with them all the time. You can't satisfy everyone (no. Y/15)** (38) **Of course, they ask. People demand to listen. We all require listening. And we also like to listen to each other. (…) Patients are the same everywhere. It is the same whether they are rich, poor, or… People demand much listening. I notice that. Do we give it to them? When we can. When we can't, we don't give it to them (no. Y/17)**
**Major theme 6: Decision making and work overload: a form of vertical power**	
**Minor theme 8: No time to decide**	**Minor theme 9: Imposition over the decision**
**Older women**	**Primary care nurses**
(39) **I hardly talk to her. I don't speak. After all, she doesn't have much time because she has… It's just that people who come like this don't give you time to talk to them because you entertain them (no. 5)** (40) **I let her do what she says. “Oops, well, today I'm not going to take your blood pressure”. She doesn't like to do that very much because she knows I have it high and I… Well, it worries me… and it raises a lot. And she has a hard time lowering it. What she does many times is not take it so as not to upset me, so as not to worry me, but she has suffered it (no. 6)**	(41) **So you have to get used to them a bit, and often it is true that the barrier, when you impose, but look, well, “This would come in handy”, for example (no. X/13)** (42) **And now you tell her, well, first of all, don't scold her, how will you scold her? And then they don't even open their houses to you again. That is the art, too, of… for you to keep opening up and see if any habit changes (no. X/13)** (43) **I believe that the changes come from that. Well, they fall, and they are the ones who find themselves with a broken hip, and the world already decides for them. They stop deciding (no. X/10)**
**Major theme 7: Participation, work overload and personal characteristics**	
**Minor theme 10: No time to participate**	**Minor theme 11: To wish is not always being able to**
**Older women**	**Primary care nurses**
(44) **I am, of course, a woman, not because I want to sell myself a little problematic, you know? I adapt to… [the situation] (no. 3)** (45) **No, because this woman [the nurse] is very busy, and you can't say… What am I going to say? If this woman… “We have a mess; such a mess…”. Of course, they all come running (no. 5)**	(46) **Sure, of course, we usually ask… (no. X/12)** (47) **We inform… They are told (…) You can try to negotiate, but they don't change much either… their behaviours and minds (no. X/11)** (48) **The objectives of Virginia [Henderson], the care plans, the individualised attention plans… It's all that they take care of themselves, themselves, but… (no. X/11)** (49) **You make her participate: at least make her aware of her limitations, of what would be suitable for her (no. X/13)** (50) **Because you can't tell them that they're doing it wrong. They have already fallen, tho. “And you tell me this?” Because you can't tell them, “This must be removed”. We also enter like this many times. “This goes out”. And you know… sometimes they listen, those fearful do listen. [she laughs] So that the nurse doesn't scold the patient when she goes back [she laughs] (no. X/13)** (51) **It´s complicated. We are talking… we are talking about communication and human relationships. So you have to be even more careful if you want habits to be changed (no. X/13)** (52) **Also, it's still an agreement between them [the older women] and us; you can't tell them everything they have to do if they don't like it because if they don't, they won't make any changes. So, you have to come to an agreement (no. X/10)** (53) **There are other things, we check vital signs, the medicine chest, “Let's see what it has”, and, come on, I don't know about your [referring the other nurses] case, but in many cases, they show you what they want you to know, and not what they really have (no. Y/14)**
**Major theme 8: Normalised subordination of the older patient**	**Major theme 9: Moral authority of the nurse**
**Minor theme 12: Asking for help is a form of abuse of power**	**Minor theme 13: Professional pride**
**Older women**	**Primary care nurses**
(54) **I don't abuse anyone or anything (no. 2)** (55) **I have nothing to do. I don't have to force her to do anything (no. 2)** (56) **No, because I tell you this, I understand that they have much work and there are very few people. Her work, you realise, that she not only works there, in the office, but then she has a home visit, another visit, another old one, another older one… (no. 4)**	(57) **Here we are, the oldest, and we´ve worked for many years. We have had an outstanding school. (…) I will not tell you that it is one of the best because it sounds pedantic, but it has given us a reasonably good job. (…) We are trying to convey this to the young nurses who have come recently (no. Y/17)** (58) It also counts not only the nurse but the team. If you're a good nurse, but then the doctor you're dealing with is a little b******, huh? Who goes to… to the nurse, meh [she makes a derogatory gesture], that throws you down a lot. (no. Y/16)
**Major theme 10: Time for giving proper attention and work overload: a frustrated wish**	
**Minor theme 14: I want them to spend more time, but they can't**	
**Older women**	
(59) **Of course, I would like that! But since she comes with… [she laughs] With that bit of time, she has to go from here to there… (no. 3)** (60) **No… I know they [the nurses] have much work. You know they've been screwed over enough by others. (no. 4)** (61) **No, not me, because the woman comes running! I open that door [she points out the entrance door] for her so she doesn´t lose time. She has to visit another, and then she has to go there… she says, “We have blood to collect today”. And as she comes, “Oh, oh! Today I come quickly, what a load I have today” (no. 5)** (62) **She has never told me, “I can't go”. No, she's coming. More time, less time. “Oh, I have to go because I'm in a hurry. I’m in a lot of…” She's always very loaded. And she consults from here to the doctor (no. 6)** (63) **She is very loaded. She is very interested [in the sense that the nurse seems interested in the older woman’s well-being], and it doesn't bother me; on the contrary, I see her… (no. 6)** (64) **Well, I understand that little angel has worked a lot… Much work. And if she must attend to several patients… “The blood, the anticoagulants, the wound care…” and so on… I don't think she has a good time for you to stop her either… (no. 9)**	

To facilitate further understanding of the phenomenon studied and its hierarchical distribution, we also calculated in Additional file 4 the magnitude of the derived findings provided by older women, primary care nurses and in common [57].

We synthesised the major themes until a higher level of qualitative meaning was found; the study patterns followed: Ideology in care, context as the axis of quality of care, a rift in communication and power imbalance in the nurse-patient relationship.

### Pattern A: Ideology in care

Primary care nurses entirely marked the first study pattern. The speech acts of the nurses showed the sociocognitive panorama that supports the bases of their work attitude, which held evident ideological traits that biased the care they provide.

### Major theme 1: Influence of ageism on care

The discursive strategies of generalisation, through the use of the syntax of quantity, aphorisms, and use of impersonal verbs; of severity intensification and polarisation, through the usage of qualifying adjectives, enumerations and repetitions showed the conception that the nurses had rooted in their cognitive heritage: older patients are a problematic patient profile to manage who do not face their age as a characteristic definition of their state of health.

(2) Older people think their problem is solved with pills (no. X/10).

*(3) It looks like the older patients, you already know that it doesn't matter if you talk to them that you already know that they come to you to prescribe pills and that it doesn't matter what you speak to them that when they leave the door, they will do what they want... (no. X/10).*

This contrasted with the projection of a public image of involvement and consideration for old age, illustrated by suggesting that older women should prevent before falling.

*(1) I have [she names an older woman], who has already fallen many times, she has a walking frame at home. I tell her to lean on it, to try… and I come another day, and she tells me she got stitches on the head because she fell. And no matter how hard you try, they don't change (no. X/10).*

The primary care nurses presented clear signs of ageism in their speech acts but also introduced the concept that the older women had an ageist attitude by not accepting advanced age.

*(4) She's not old, and you tell her something. She says, “that's because I'm old?”, what if I take the walking frame, am I old? And she is 90 years old, but that is your security, it is her security, so then… (no. X/10).*

The primary care nurses also established a difference in approach between young and older patients, anticipating the possible needs and declaring that since young people deal with more information, any health issue should be addressed more clearly with them than older patients.

(8) Many young patients with wound care have already seen what is good and what is not. Now you have to be more… tactful, in explaining the procedure (no. Y/17).

#### Major theme 3: Influence of gender on care

Firstly, the conception of gender had an essential impact on the ideology of care when primary care nurses, in the first place, presupposed that the responsibility for a male older diabetic patient´s health lay partly with his wife because she was responsible for taking care of his meals.

(16) When a diabetic man comes *here*, I usually tell him, tell him to come with his wife too, because she is also the one who… (no. X/10).

(17) [Referencing (16)] Who is the one who cooks the meal for him (no. X/13).

Secondly, the nurses also declared that they developed paternalistic behaviours and excessive protection of their older patients. Their discursive strategy focused on adopting a public image of a victim —in the face of an incessant demand by older patients—using a lexicon with clear ideological implications.

(18) But maybe I have a more paternalistic attitude than my colleagues, and I would often like to get rid of it, but I don't know. I don't know why I don't know how to say no to people either. Everything they blame me, they trick me, and I say yes. So that leads to much sleep being taken away, but oh well. So there are things that I know I'm not doing well (no. Y/17).

### Pattern B: Context as the axis of quality of care

The study phenomena comprised in the objectives of this study gained strength by giving shape to the second study pattern. The findings not only revealed deficiencies in nursing care in situations of AL, SDM and participation, but also the nursing labour context became highly relevant when misaligning the attitude and approach of primary care nurses regarding managing such situations.

### Major theme 5: Active listening and work overload: a privilege

Older women´s feeling of not being listened to was due to primary care nurses' work overload. The speech acts that indicated this gained an illocutionary force that seemed to create a public image of older women as another number in the primary care nurses´ services.

(34) She says she also has to attend to many people. She says, “It's just not you alone, but… it's another, another, another. One day I dedicate to one, another day I dedicate to another” (no. 2).

On occasions, the older women, using apparent concessions, attempted to persuade by introducing a positive component in favour of the nursing collective to create a non-negative public image. Then, they followed with a contradictory statement.

(35) Of course, I don't think she [the nurse] won't listen to me. That everyone listens, right? Although later they say, look, this is how it is, it is like that, and it's over (no. 5).

The older women maintained the cognitive assumption that they had the right to be listened to and that the nurse had the duty to listen to them.

(36) Even if she doesn’t want to, she has to listen to me, whatever I tell her, right? And she comes running, and maybe she can stop; if I talk to her about anything, the woman should listen to me (no. 5).

These assumptions collided with the speech acts of the primary care nurses, who did not fulfil older women’s desires to be heard: these suggested two different implicatures. First, if fewer patients were to be attended, the primary care nurses could dedicate more time to listen to older women; second, everyone deserves to be heard, and therefore nurses also deserve to be heard, this being denied and ignored on numerous occasions. Thus, primary care nurses presented themselves as a social group that was also vulnerable, and little listened to, ousted by the inefficient organisation of care and work overload.

(37) It´s not always possible because there are many, and they want you to be with them all the time. You can't satisfy everyone (no. Y/15).

*(38) Of course, they ask. People demand to listen. We all require listening. And we also like to listen to each other. (…) Patients are the same everywhere. It is the same whether they are rich, poor, or… People demand much listening. I notice that. Do we give it to them? When we can. When we can't, we don't give it to them (no. Y/17).*

### Major theme 6: Decision making and work overload: a form of vertical power

The older women eclipsed the opportunity to decide when they declared not to talk to the nurses.

(39) I hardly talk to her. I don't speak. After all, she doesn't have much time because she has… It's just that people who come like this don't give you time to talk to them because you entertain them (no. 5).

There was a case of a pact of silence between the primary care nurse and the older woman, where the first opted not to carry out some intervention, such as a blood pressure check. The older woman was grateful that this decision was made due to her fear of explicitly knowing her state of health. This did not represent SDM: the nurse decided not to check the blood pressure promptly because she assumed it would be high and, in turn, generated concern in the older patient. In this sense, older women displayed examples like this to extol the commitment of primary care nurses to be sensitive to their preferences and mask an evasion of SDM, which was reinforced through repeated syntax parallelisms with great illocutionary force.

*(40) I let her do what she says. “Oops, well, today I'm not going to take your blood pressure”. She doesn't like to do that very much because she knows I have it high and I… Well, it worries me… and it raises a lot. And she has a hard time lowering it. What she does many times is not take it so as not to upset me, so as not to worry me, but she has suffered it (no. 6).*

This is why the older women perceived primary care nurses as empathic and compassionate throughout the discourse. Still, at the same time, there was a lack of equality since the imbalance in decision-making was total.

On the other hand, this was easily seen when we turned our gaze to primary care nurses´ discourse, who used a lexicon with a pragmatic charge of domination and the use of verbal structures with an oppressive connotation.

(41) So you have to get used to them a bit, and often it is true that the barrier, when you impose, but look, well, “This would come in handy”, for example (no. X/13).

*(42) And now you tell her, well, first of all, don't scold her, how will you scold her? And then they don't even open their houses to you again. That is the art, too, of… for you to keep opening up and see if any habit changes (no. X/13).*

Furthermore, the discourse of the primary care nurses resounded in that the ability to decide did not correspond to older women but rather to their illness circumstances and health conditions.

(43) I believe that the changes come from that. Well, they fall, and they are the ones who find themselves with a broken hip, and the world already decides for them. They stop deciding (no. X/10).

### Major theme 7: Participation, work overload and personal characteristics

The illocutionary speech acts of older women sought to convey a public image of agency and collaboration. For this purpose, they used the discursive resource of negation with an inverse effect. By making explicit what they did not want to do, they achieved precisely the opposite, so the intention to underline that they were not very problematic achieved that goal.

(44) I am, of course, a woman, not because I want to sell myself a little problematic, you know? I adapt to… [the situation] (no. 3).

Older women discarded the possibility of participating in their care by embracing nurses’ workload because they did not have the opportunity to do so. The speech acts of the older women were hasty, disconnected and unfinished, so the primary care nurses' discourse influenced older women´s cognitive model of active participation in care. The older women assumed that the only way to participate was through a circumstance beyond their control: the workload of the primary care nurse who cared for them.

(45) No, because this woman [the nurse] is very busy, and you can't say… What am I going to say? If this woman… “We have a mess; such a mess…”. Of course, they all come running (no. 5). In other words, older women considered themselves subdued to a context that did not allow them to participate.

The power relationship this time transcended the figure of the primary care nurse to reach the health system organisation. The primary care nurses’ statements once again clashed with those of the older women, saying they were assertive and facilitated the participation of older women.

(47) We inform… They are told (…) You can try to negotiate, but they don't change much either… their behaviours and minds (no. X/11).

The nurses based their inclusive actions on informing older women and understanding how to make them participate by making them aware of their health and, therefore, of their limitations.

(49) You make her participate: at least make her aware of her limitations, of what would be suitable for her (no. X/13).

The primary care nurses exercised discursive strategies of generalisation and exemplification, positioning themselves as professionals capable of involving older women in the care processes. However, there were difficulties when older patients did not wish to be engaged.

*(52) Also, it's still an agreement between them [the older women] and us; you can't tell them everything they have to do if they don't like it because if they don't, they won't make any changes. So, you have to come to an agreement (no. X/10).*

*(53) There are other things, we check vital signs, the medicine chest, “Let's see what it has”, and, come on, I don't know about your [referring the other nurses] case, but in many cases, they show you what they want you to know, and not what they really have (no. Y/14).*

### Major theme 10: Time for giving proper attention and work overload: a frustrated wish

There was an imbalance between older women´s needs and compliance: here, a desire to receive more care predominated, but once again, due to the primary care nurses´ work overload, that desire was frustrated. This implicature was supported using illocutionary, exclamatory statements and conversational resources that relativised seriousness, such as laughter, and the rephrasing of arguments already used by the nurses to intensify their views.

(59) Of course, I would like that! But since she comes with… [she laughs] With that bit of time, she has to go from *here* to there… (no. 3).

Older women did not explain why they would like the primary care nurses to spend more time with them: they argued why they presuppose that the nurses did not spend that time instead. This implied that having little time was something nurses did not want, so the locus of power fell neither on the older women nor the nurses but on those who “screw over”.

(60) No… I know they [the nurses] have much work. You know they've been screwed over enough by others. (no. 4).

*(61) No, not me, because the woman comes running! I open that door [she points out the entrance door] for her so she doesn´t lose time. She has to visit another, and then she has to go there… she says, “We have blood to collect today”. And as she comes, “Oh, oh! Today I come quickly, what a load I have today” (no. 5).*

The power relationship here was once again translated into healthcare organisational elements framed in the following ranks: firstly, those who “screw over” or, in other words, the people in charge, which prevented primary care nurses from the opportunity to spend more time with older women; secondly, the nurses who through their speech acts, annulled the possibility of reversing this situation; thirdly, older women, who saw their desire to receive more care time double denied by both the first and the second ranks.

The speech acts of the older women revealed a differentiation in the care they received: one thing was receiving a visit from the primary care nurse, and another was the time spent during that visit. Regarding the second, older women did not clarify whether the nurse spent much or little time caring for them in the first instance. Still, throughout the discourse of an older woman, there was the presupposition of illocutionary force accompanied by a metaphor that the nurses spent insufficient time.

*(64) Well, I understand that little angel has worked a lot… Much work. And if she must attend to several patients… “The blood, the anticoagulants, the wound care…” and so on… I don't think she has a good time for you to stop her either… (no. 9).*

However, there was another illocutionary effort by the older women to exonerate primary care nurses from their inefficient praxis at the same time; that is, older women tried to make public the assumption that the professionals who cared for them were not only interested in caring but also met older women´s necessities. These presuppositions acquired great perlocutionary force since the older women sometimes identified an almost admiration feeling for the commitment they perceived from the primary care nurses.

(62) She has never told me, “I can't go”. No, she's coming. More time, less time. “Oh, I have to go because I'm in a hurry. I’m in a lot of…” She's always very loaded. And she consults from *here* to the doctor (no. 6).

(63) She is very loaded. She is very interested [in the sense that the nurse seems interested in the older woman’s well-being], and it doesn't bother me; on the contrary, I see her… (no. 6).

With all this, it was just a confirmation that the nurses —in the eyes of the older patients— were very busy, but even so, they showed interest in caring for them.

### Pattern C: A rift in communication

The third study pattern represented a communicative interference in which complications constituted by personal characteristics from the older adults and the primary care nurses and the lack of disposition and communicative attitude cracked a fissure between the relationship both maintained during nursing care. These non-therapeutic situations could be saved numerous times if a more beneficial healthcare context supported a more balanced and horizontal approach between equals.

#### Major theme 2: Absence in the organisation of care

Older women sometimes found it difficult to argue in favour of nurses’ good work in managing visits and time slots. Discursive strategies that intensify insecurity, such as silences, hesitant language, and zigzagging speech acts, hinted at discomfort with the questions raised.

*(12) No, sometimes she comes because she had to; she always had a fixed day, the last two days of the month she always came. And she no longer called; she showed up… Oh, [she names the nurse], how are you coming and not… “Don´t you know already that I come these days?” (no. 6).*

There were times when older women found themselves at a crossroads that they presupposed did not correspond to them when they considered whether to attend to leisure needs or instead a sudden visit that had not previously agreed with the primary care nurses. These situations were visibly uncomfortable in the speech acts of an older woman using syntax constructions that served as conflict intensifiers.

*(9) Well… they call me. And I have an appointment with the hairdresser. What do I tell the hairdresser now? That I'm not going? And what do I say to the nurse? Do not come? [she laughs] Yeah, I question, I call into question (no. 1).*

However, after raising these dilemmas with insecurity, a resounding reversal in her speech acts —more concise and direct—gave way to an apparent decision. The older woman decided on that occasion to dispense with the unscheduled visit by the primary care nurses.

*(10) [Interviewer asks referencing (9): and what do you do?] Well, go to the hairdresser's, because I feel fine and don't need the nurse right now… And I need the hairdresser's because I'm going out. And I decide to go to the hairdresser (no. 1).*

It was notorious how the older woman defended this with assertive, clear and confident speech acts of illocutionary force and acquiring an image of a role of power and choice. However, this could be considered a mirage of a decision-making situation since she believed she was deciding but was forced to resolve something previously imposed by a previous decision of the nurses —not reconciling with any other activity previously scheduled by the older woman—. Therefore, the primary care nurses held a substantial role of power in that situation.

The discursive strategies used by older women also acquired a metaphorical component with considerable pragmatic and illocutionary force.

(11) No, the nurses come directly, and if I'm not there, well, the little angels leave. I can't demand an hour from them because they have much work (no. 4).

This resource fulfilled two functions: first, to show a compassionate attitude free of locus of power, thus renouncing the possibility of coordinating with the primary care nurses; the second, to create a public image of tenderness and a docile patient who feels distressed when the nurses—even without having previously consulted with older women about visiting hours—went to their homes and were not present. The responsibility, therefore, fell on the older women for not being at home and not on the nurses.

Although older women made their preferences explicit, sometimes they did not communicate them to the primary care nurses.

(13) [Referencing (12): and do you tell the nurses what you prefer…?] Well, I´d prefer that she reports me because since I'm alone, sometimes I'm in a housecoat, sometimes I'm… [she laughs]. (no. 6).

Hence, their role as passive patients emerged at that moment: they felt uncomfortable when they were not notified —actually, the older women *preferred* to be informed—but did not express that discomfort.

### Major theme 4: Ineffective communication: opposing attitudes

Regarding the deficit of trust with the nurses, the speech act where an older woman tried to show power to decide when she introduced a relative as a communicative alternative was striking. Not sharing thoughts with the nurses represented the older woman´s verbal regret for not doing so.

(19) If I feel bad, I count on my sister. That´s it. I have more confidence in her (no. 1).

(20) I say many times, I think for myself. (…) I think so. I think about it to myself, but not to… tell anyone (…) But, yes, I had to say to the nurse (no. 1).

The older women tried expressions that sought to relieve the seriousness of a non-therapeutic communicative situation, such as using rhetorical question resources accompanied by laughter, thus establishing a tone of certain lightness and minor importance.

(22) It seems… I don't care. But since I have no problems… What do I say? [she laughs] (no. 2).

On the other hand, there was a case in which the stylistic expression gained remarkable strength since it had such an illocutionary charge, displacing the locus of action to an inanimate subject with a verbal form lacking agency.

(23) I tell her [the nurse] often that the afternoons seem very long for me (no. 2).

This made us see that there are times when older women felt lonely and lacked the power to reverse those situations of loneliness, despite communicating this time to the primary care nurses. This expression had a metaphorical, abstract background, giving a natural phenomenon such as the passage of time the ability to shape the real world of older women, having an intention prone to creating compassion and empathy.

Throughout the various speech acts of the older women, there was also an illocutionary force that made us detect a state that crossed the line between not communicating for lack of necessity and not communicating because they considered themselves a burden, representing themselves as a nuisance for the performance of home nursing tasks.

(24) It makes me very tired of that. It is because I say the nurses have their work, they have their check-ups, their things and I am not going to spoil them (no. 3).

(25) I don't want to bother my children either. And when something hurts me half the time, I don't even tell them. (…) Because I don't want them to suffer! I'm suffering a lot… (no. 3).

Regarding the discourse of the primary care nurses, they felt pointed out by the older patients and in a blind alley since they stated that on certain occasions, they were unsure how to act due to possible adverse reactions from the older patients and relatives. In those speech acts, they used rhetorical-stylistic resources such as rhetorical questions and conversational resources such as laughter to lower the tone and reach complicity.

*(29) Many say, “their nurse had to tell the patient to wash”. And you say… “let's see. Do I tell him…? How do I tell him?” Well… Sometimes… and if you tell him, he gets offended. And the wife asks for another nurse because she says I made her see that she did not take good care of her husband (no. X/11).*

*(30) You go to their house, and that´s tough already. “Where does that carpet come from?” They tell you the story of that carpet. That she brought it from… “Excuse me? You´re going to take it away? Why? This furniture… what? My husband and I put it up, this piece of furniture here isn´t going anywhere…”. But look, the walking frame does not fit. Well, I'm left without a walking frame! But I don't remove that piece of furniture or the carpet [she laughs] (no. X/11).*

The nurses' discourse in these terms tried by all means to illustrate, through implicatures, the frustration they sometimes felt when they wanted to seek an improvement in the health status of older women and, at the same time, could not avoid slipping the preconception that everything happens according to what older people want at the moment, giving older people some power. However, this contrasted strongly with the discourse of older women, who attributed the ability to act outside of themselves.

### Pattern D: Power imbalance in the nurse-patient relationship

In the last study pattern, we were glaringly able to detect the power imbalance between older women and primary care nurses. From a socio-cognitive point of view, the speech acts of both denoted a discriminatory conception of care embedded in their mental processes, where the patient was someone who receives and does not demand; the care provider was someone who knows and, therefore, an indisputable authority in her field of action.

### Major theme 8: Normalised subordination of the older patient

The older women conveyed the assumption that asking the primary care nurses to perform some extra activity was an abuse of power. In these cases, older women saw themselves in a position of power for merely receiving nursing services. Instead of becoming a legitimate resource they could use as needed, this became a self-limiting resource: older women assumed that nurses should not adapt to their individual needs, relegating their health states one step lower to avoid interfering with the activities the nurses carried out systematically.

(54) I don't abuse anyone or anything (no. 2).

(55) I have nothing *to do*. I don't have to force her *to do* anything (no. 2).

This made more sense when they attributed not asking for help to the primary care nurses’ work overload.

*(56) No, because I tell you this, I understand that they have much work and there are very few people. Her work, you realise, that she not only works there, in the office, but then she has a home visit, another visit, another old one, another older one… (no. 4).*

However, this seemed to be a discursive strategy directly influenced by a circumstance acquired by two different possibilities: first, it was evident that the older patient could not know the real labour context of a particular nurse unless that nurse had told her previously; the second, it was their presupposition, that is, they considered that nurses did not have time to attend to their extra needs.

### Major theme 9: Moral authority of the nurse [57, 58]

Finally, we identified in the discourse of the primary care nurses that age —when they referred to themselves— was an indicator of wisdom and moral authority, something that suddenly contrasted when they talked about advanced age in the case of the older women, according to the ageist attitude displayed in study pattern A.

*(57) Here we are, the oldest, and we´ve worked for many years. We have had an outstanding school. (…) I will not tell you that it is one of the best because it sounds pedantic, but it has given us a reasonably good job. (…) We are trying to convey this to the young nurses who have come recently (no. Y/17).*

In addition to the veiled intentionality in the primary care nurses’ speech acts, the lexicon used denoted pride when referencing their training as the best possible, which they tried to convey to younger nurses.

In addition, they closed ranks around the nursing team, placing the medical figure on the balance of multidisciplinary, sometimes prone to question the authority and power of nurses.

(58) It also counts not only the nurse but the team. If you're a good nurse, but then the doctor you're dealing with is a little b******, huh? Who goes to… to the nurse, meh [she makes a derogatory gesture], that throws you down a lot. (no. Y/16).

Therefore, the speech acts of the nurses showed us a sharp sense of the social group as an essential component in their professional identity and an emblem of their category of power towards older women, sometimes questioned by other teammates. Thus, not only an imbalance of power between social groups was detected, female older patients and primary care nurses, but also possible intra-social group disagreements between nurses and doctors.

## Discussion

The results of this study revealed that, above all, age discrimination remains silent in society, especially in health care [58]. The primary care nurses in our study diverted the ineffective results of their health interventions towards an attitude of the older women with ageist overtones and a lack of commitment. This agrees with Tajfel's Social Identity Theory [59], which considers the attitude of different social groups as a reflection of the identity of belonging to a group, which tries to make their excellent work visible above others, over which they stand out other negative aspects [32, 51]. The discourse of the older women also revealed that they had self-ageist conceptions, which is consistent with Levy's Stereotype Embodiment Theory (SET) [60], which describes how older people internalise a negative self-perception of ageing (SPA) by incorporating ageist signals that society performs subtly. On the other hand, gender ideology was also present in the results concerning primary care nurses, who sometimes assumed paternalistic behaviours that prevented them from refusing any request from older people. Therefore, the discursive victimisation strategy used by nurses tried to take a positive public image, basing care as a labour of love and sacrifice rather than professional competence. This was consistent with what was exposed by Saillant [61], who postulates the concept of caring as a gender issue and a direct consequence of unbalanced power relations between the sexes [62]. The nursing care processes contemplated in our study's objectives greatly impacted older women's discourses, becoming the epicentre of the imbalance in power relations. This importance of reconciling AL, decision making and participation in older people is consistent with what Koskenniemi et al. [63] maintained as the pillars that the older people themselves defined when referring to care based on respect: acceptance, AL, engagement and warmth in care [64]. However, in the present study, the discourse of older women diminished the appearance of AL processes due to the unavailability of primary care nurses. These results are consistent with other studies that point to a poor attitude on the part of nurses and the importance of this in terms of the inclination to listen to older patients and take their preferences seriously [65, 66]. On the other hand, from the point of view of the primary care nurses' discourse, there was a component of stress and impotence due to the lack of sufficient opportunities that would allow them to develop AL towards all older patients, something consistent with the results of other studies [67, 68]. Regarding SDM situations, in the results of this study, it was readily apparent that, despite appearing sensitive moments for older women to decide, they did not materialise in real decision-making situations. This is consistent with other studies that consider this process a phenomenon full of uncertainty that requires an attitude prone to dialogue on the part of older patients and health personnel [69, 70]. On the other hand, the discourse of primary care nurses presented clear signs of power imbalance in which they adopted a dominant role over older women, being consistent with other studies, which highlight the feeling of disempowerment in older people when it occurs [71], especially in the case of older women [13]. Regarding active participation in care, the primary care nurses in our study seemed to know communication tools to engage older women to participate in their care. Still, due to the personal characteristics of the older women, this task was not easy. This is consistent with other studies that describe a perception of health personnel who also classify older people as stubborn and complicated in the attempt to include them in care processes [72, 73]. All these moments, related to situations of AL, SDM and participation in care, were plagued with anomalies that did not allow older women to take an active agency role in their care, nor did primary care nurses establish therapeutic communication with them. However, one of the star elements responsible for such interference in the relationships between primary care nurses and older women was the work overload suffered by nurses. These circumstances, present at all times, are consistent with numerous studies that point to working conditions as causing, in part, the power imbalance between the two and a decline in the quality of care provided by health personnel in primary care settings [74–76]. This work overload, as reflected by Lindberg et al. [77] in their research, could explain the fact that nurses were sometimes forced to make home visits not previously agreed upon with older women, directing the power imbalance described by the older women’s discourses not so much towards interpersonal issues, but to organisational issues. Regarding the imbalance imposed in the roles of power between primary care nurses and older women, paradoxically, there is a certain contrast between the results of the present study and the work carried out by Juujärvi et al. [78], who identified older patients as overly demanding when asking for favours and giving orders, as well as treating nurses as if they were servants. The older women in the present study did not request extra help and interpreted this as an abuse of power from their patient role and not a right. This could also be explained by the SET, through which they considered themselves a burden or a nuisance, sentiments also reflected in speech acts from the older women in this study [79, 80].

### Strengths and limitations of this study

The main strength of this study lies in incorporating the socio-critical perspective of CDA into the context of qualitative research in health sciences, specifically in the nursing discipline. The CDA has already proven helpful in nursing inquiry, as Powers [81] proposed. The study of speech acts and the pragmatics of communicative acts is an effort with great rewards in researching situations where communication is the axis of care [82, 83]. On the other hand, we have invested a great effort in describing in detail the methodological framework of this study, in addition to adequately organising the results obtained, providing in-depth information on the data collection process and following criteria of rigour and quality.

The limitations of this study address the decision to focus our study on older women who lived alone because it is associated with vulnerability in advanced age. Recent studies linked a negative SPA with loneliness, isolation, and depressive symptoms [84, 85]. This evidence has affected our results, leading to a negative SPA. Other older women in more varied social circumstances could have provided some variation concerning SPA. On the other hand, there were three interviews with older women in which their formal caregivers were also present. This fact could influence her speech somehow since the presence of third parties might modify the communicative situation. Another limitation of this study may reside in the size of the corpus analysed, being excessively large compared to the textual extracts that are usually handled in other disciplines far from nursing and analytically framed in the CDA. Due to the deep and extensive degree of detail held by microanalysis of language planes, it was unfeasible for logistical and time reasons to apply it to this study. For this reason, we decided to focus on the macroanalysis of the planes previously mentioned in the methodology section. Not for this reason, the principles of CDA agree perfectly with what was stated by Malterud et al. [86], who stipulate that in qualitative research, a sample rich in terms of information requires a low number of participants to confer it.

### Relevance to clinical practice and healthcare policies

The present study has revealed an imbalance in power relations between primary care nurses and older women. The negative effect of this imbalance on situations of AL to preferences, SDM, and participation in care reveals an urgent need to develop measures and interventions that promote a horizontal collaboration relationship between primary care nurses and older women. Implementing alternative response models to the health problems of older people in the community setting so that it is possible to include them in the decision-making processes and participation in care is vital to providing quality nursing care [87]. On the other hand, a detrimental context in which primary care nurses find themselves has been highlighted, which causes a work overload whose effects are well-known and supported by the scientific literature, negatively impacting the quality of care. For this reason, it is urgent to attend to the needs of nurses and to help the power imbalance between primary care nurses and healthcare organisations.

## Conclusion

This study´s results present contradictions between the perception that primary care nurses and older women have about situations of AL to preferences, SDM, and participation in nursing care. The discourse of older women shows a self-awareness of subordination in the face of the primary care nurse's figure and their work overload context. This concept of subordination harms communication with professionals, which is often non-existent. Older women hardly share moods or preferences due to a lack of confidence or because they consider themselves a nuisance or even a burden to primary care nurses´ performance. They declare that they are not part of any decision-making process regarding managing their care and consider their participation a possible source of problems regarding their relationship with the primary care nurses. The discursive strategies used by older women to build their social representation were those of victimisation, insecurity intensifiers and stylistic compassion. On the other hand, the discourse of primary care nurses presents clear ideological overtones that tarnish the quality of their care: ageism is present in their socio-cognitive mental schemes regarding their definitions of an older female patient; their paternalistic attitudes make them act in an overprotective and self-sacrificing manner, according to their speech acts. Primary care nurses adopt a role of power through a morally authoritarian group identity, which places them symbolically above older women. The context in which primary care nurses find themselves negatively influences carrying out their work as they would like, generating frustration and a feeling of not being heard, something that they consider an eye for an eye regarding the AL of older women. The discursive strategies used by this group to create their social representation were very varied, being those of generalisation, exemplification, victimisation, and severity intensifiers, showing a wide range that denotes that they are a group with easy access to discursive structures to reproduce their social image through speech acts.

### Supplementary Information

Supplementary Material 1. 

Supplementary Material 2.

Supplementary Material 3.

Supplementary Material 4.

## Data Availability

The data supporting the findings of this study are available at reasonable request from the corresponding author. The data are not publicly available due to privacy or ethical restrictions.

## Abbreviations

CDA: Critical discourse analysis
SPT: Social practice theory
SPA: Self perception of ageing
AL: Active listening
SDM: Shared decision-making
