# Supplementary material for: Active listening, shared decision-making and participation in care among older women and primary care nurses: a critical discourse analysis approach from a gender perspective
Source: BMC Nurs. 2024 Jun 17;23:401. doi: 10.1186/s12912-024-02086-6 (PMC11181639; doi:10.1186/s12912-024-02086-6)
Supplement: Supplementary file 1 — Supplementary Material 1. [file 12912_2024_2086_MOESM1_ESM.docx]

**Additional file 1.** Reporting of qualitative research studies. Adapted from O´Brien et al. [40].

| **Items** | **Page and paragraph numbers stated for each reporting element** |
| --- | --- |
| **Title and Abstract** | |
| 1. Title | Page 1; Paragraph 1 |
| 2. Abstract | Page 2; Paragraph 1 |
| **Introduction** | |
| 3. Problem formulation | Page 4; Paragraph 1 |
| 4. Purpose or research question | Page 5; Paragraph 3 |
| **Methods** | |
| 5. Qualitative approach and research paradigm | Page 6; Paragraph 1 |
| 6. Researcher characteristics and reflexivity | Page 8; Paragraph 4 |
| 7. Context | Page 6; Paragraph 3 |
| 8. Sampling strategy | Page 6; Paragraph 4 / Page 8; Paragraph 1 |
| 9. Ethical issues pertaining to human subjects | Page 10; Paragraph 2 |
| 10. Data collection methods | Page 8; Paragraph 3 |
| 11. Data collection instruments and technologies | Page 9; Paragraph 2 |
| 12. Units of study | Page 8; Paragraph 4 / Page 11 Paragraph 1 |
| 13. Data processing | Page 8; Paragraph 4 |
| 14. Data analysis | Page 9; Paragraph 3 |
| 15. Techniques to enhance trustworthiness | Page 11; Paragraph 1 |
| **Results/findings** | |
| 16. Synthesis and interpretation | Page 12; Paragraph 1 |
| 17. Links to empirical data | Page 12; Paragraph 4 |
| **Discussion** | |
| 18. Integration with prior work, implications, transferability, and contribution(s) to the field | Page 21; Paragraph 1 |
| 19. Limitations | Page 28; Paragraph 2 |
| **Other** | |
| 20. Conflicts of interest | Page 31; Paragraph 4 |
| 21. Funding | Page 31; Paragraph 5 |
