# Supplementary material for: Active listening, shared decision-making and participation in care among older women and primary care nurses: a critical discourse analysis approach from a gender perspective
Source: BMC Nurs. 2024 Jun 17;23:401. doi: 10.1186/s12912-024-02086-6 (PMC11181639; doi:10.1186/s12912-024-02086-6)
Supplement: Supplementary file 2 — Supplementary Material 2. [file 12912_2024_2086_MOESM2_ESM.docx]

**Additional file 2.** Reporting qualitative research interviews and focus groups. Adapted from Tong et al. [41].

| **Domain 1: Research team and reflexivity** | **Researcher response** |
| --- | --- |
| **Personal Characteristics** | |
| 1. Interviewer/facilitator  Which author/s conducted the interview or focus group? | PMA conducted all the interviews. |
| 2. Credentials  What were the researcher’s credentials? | PMA was a PhD student and MSc in Health sciences research. MRR, MRJM, and SLQ were PhDs. |
| 3. Occupation  What was their occupation at the time of the study? | PMA was working as a Research Fellow at the University of Córdoba (UCO). MRR and SLQ were working as full professors at UCO. MRJM was working as an assistant professor at UCO. |
| 4. Gender  Was the researcher male or female | PMA, MRR, and SLQ are male. MRJM is female. |
| 5. Experience and training  What experience or training did the researcher have? | PMA had experience in qualitative research from previous works. He received a Master’s degree in Health sciences research from the University of Jaén (UJA), Spain. In addition, PMA has received training in advanced doctoral seminars on CDA and holds a postgraduate certificate in Masculinities, Gender and Equality from the Miguel Hernández University (UMH). He also gave teaching lectures during his fellowship. |
| **Relationship with participants** | |
| 6. Relationship established  Was a relationship established prior to study commencement? | There was no previous relationship with any interviewee since the interviewer of the research team knew them at the time of data collection. PMA had a prior relationship with the rest of the group. MRR and SLQ were their doctoral thesis supervisors; MRJM was a colleague from the department. |
| 7. Participant knowledge of the interviewer  What did the participants know about the researcher? e.g. personal goals, reasons for doing the research | PMA informed the participants that the research project belonged to a thesis project of PMA. The researcher told the older women and primary care nurses that his purpose was to discover how situations of active listening, shared decision-making and participation in care developed within older women-primary care nurse relationships. When the participants asked questions about the project, they were answered by both researchers. |
| 8. Interviewer characteristics  What characteristics were reported about the interviewer/facilitator? e.g. Bias, assumptions, reasons and interests in the research topic | The principal interest of PMA in the topic was based on his desire to focus the thesis project and future research on situations of social injustice, imbalance of power, and ageism in care contexts in the primary care setting. |
| **Domain 2: Study design** |  |
| **Theoretical framework** | |
| 9. Methodological orientation and Theory  What methodological orientation was stated to underpin the study? e.g. grounded theory, discourse analysis, ethnography, phenomenology, content analysis | The research paradigm for this study was sociocritical from a discursive and gender perspective. Using the CDA framework, the researchers had an approach to possible social injustice and discrimination to locate care failures and highlight areas for improvement in the care from health services. Having a gender perspective, the researchers could have an insight into how these power relationships are shaped in older women and female primary care nurses. This study analyses the power relationships between older women living alone and primary care nurses attending them at home. We employed linguistic analysis concerning discourses to understand older women and primary care nurses´ interpretations of the phenomena studied. |
| **Participant selection** | |
| 10. Sampling  How were participants selected?  e.g. purposive, convenience, consecutive, snowball | The article explained the sampling method, and all approached older women and primary care nurses agreed to participate. |
| 11. Method of approach  How were participants approached?  e.g. face-to-face, telephone, mail, email | All study participants were approached face-to-face with help from nurse care managers and referral nurses who reached the older women. In the case of primary care nurses, a face-to-face approach was held with assistance from nurse care managers. |
| 12. Sample size  How many participants were in the study | In total, nine semi-structured interviews and two focus groups were conducted. The interviewees were older women who lived alone in their homes; primary care nurses who attended to older women with nursing home services. |
| 13. Non-participation  How many people refused to participate or dropped out? Reasons? | None of the participants who were asked to participate refused to be part of the study or withdrew from it at any time. |
| **Setting** | |
| 14. Setting of data collection  Where was the data collected? e.g. home, clinic, workplace | The interviews took place at the participant’s preferred location. This location was their home for older women. Indoor and conditioned enclosures in their respective health centres regarding primary care nurses. |
| 15. Presence of non-participants  Was anyone else present besides the participants and researchers? | Caregivers were also present at the interviews with participants 4, 6, and 7. |
| 16. Description of sample  What are the important characteristics of the sample? e.g. demographic data, date | All participants´ characteristics are described in Tables 2 and 3. |
| **Data collection** | |
| 17. Interview guide  Were questions, prompts, guides provided by the authors? Was it pilot tested? | The authors provided the interview guides as additional files to this article. Considering the dynamic nature of the focus groups, the interview guide topics were used as a starting point for them. The list of topics was adjusted to each situation´s uniqueness throughout the research interview phase. |
| 18. Repeat interviews  Were repeat interviews carried out?  If yes, how many | Repeated interviews with the participants were not conducted. As for the patients, this was due to their multimorbidity and because many reported their state of fatigue concerning the realisation of interviews. As for the primary care nurses, this was due to their difficulties in joining again. |
| 19. Audio/visual recording  Did the research use audio or visual recording to collect the data? | All interviews were audio recorded with the participant’s verbal and written consent. The recordings were stored on PMA computers because he was responsible for data analysis. Only he had access to this data. |
| 20. Field notes  Were field notes made during and/or after the interview or focus group? | The audio recording was accompanied by a reflective field notebook which included observations and impressions that were not recorded, such as the non-verbal communication of the participant. It contained reflections through a self-hermeneutic process during the study as well. Field notes were used in the analysis of the results afterwards. |
| 21. Duration  What was the duration of the interviews or focus group? | The time of the semi-structured interviews was approximately 60 minutes on average. The time of focus group X was 50. The time of focus group Y was 70 min. |
| 22. Data saturation  Was data saturation discussed? | Data saturation was discussed with the research team. |
| 23. Transcripts returned  Were transcripts returned to participants for comment and/or correction? | Due to various reasons (such as the limitations in the reading of most of the patients due to medical or literacy issues; a state of exhaustion after the completion of the interviews), the transcripts were not returned to the older women for comments or feedback. The transcriptions were not returned to the primary care nurses because of unavailability. |
| **Domain 3: Analysis and findings** |  |
| **Data analysis** | |
| 24. Number of data coders  How many data coders coded the data? | PMA performed the CDA. SLQ supervised the analysis alongside MRR. |
| 25. Description of the coding tree  Did authors provide a description of the coding tree? | No coding tree was used. The themes were derived from the CDA. The authors provided narrative and visual development of this process in the article. |
| 26. Derivation of themes  Were themes identified in advance or derived from the data | The themes were derived from the data and were discussed and agreed on by  all the authors. |
| 27. Software  What software, if applicable, was used to manage the data? | SimpleMind Pro was the software tool for managing the data visually. |
| 28. Participant checking  Did participants provide feedback on the findings? | Due to several reasons, as explained at number 23, there was no feedback from the participants on our findings after the interviews or focus groups. |
| **Reporting** | |
| 29. Quotations presented  Were participant quotations presented to illustrate the themes/findings? Was each quotation identified? e.g. participant number | The results section is illustrated with speech acts as quotes from the participants. Each quote is identified with a participant number. |
| 30. Data and findings consistent  Was there consistency between the data presented and the findings? | According to our assumption, the data presented in the study and the results that emerge from them are consistent. |
| 31. Clarity of major themes  Were major themes clearly presented in the findings? | The major themes are present in the results section of our article. Each theme is assigned a different heading. |
| 32. Clarity of minor themes  Is there a description of diverse cases or discussion of minor themes? | The minor subthemes are described along with the major themes, accompanied by specific quotes for each one. |
