# Supplementary material for: Active listening, shared decision-making and participation in care among older women and primary care nurses: a critical discourse analysis approach from a gender perspective
Source: BMC Nurs. 2024 Jun 17;23:401. doi: 10.1186/s12912-024-02086-6 (PMC11181639; doi:10.1186/s12912-024-02086-6)
Supplement: Supplementary file 3 — Supplementary Material 3. [file 12912_2024_2086_MOESM3_ESM.docx]

**Additional file 3a.** Interview guide for older women.

***Preliminary questions***

1. How has your experience with nursing home care been?
2. Do you think they somehow treat you differently because of your age?
   1. [Yes] In which terms?
   2. [No] Why not? **Reasons**.

***Appointments***

1. When arranging an appointment, do nurses take you into account to establish the time slot for visits? [*care organisation*]

***Nursing care services***

1. Do you think the nursing care services you receive are those you really need? [*convenience*]
   1. [Yes] Do you receive those nursing care services when you need them? [*timely*]
   2. [No] Why not? **Reasons**.
2. Have you expressed your opinion regarding the nursing care services you receive or the treatment you take?
   1. [Yes] In which situations?
   2. [No] Why not? **Reasons**.

***Nursing home visits***

1. Do the nursing home visits adapt to your daily life/ lifestyle? [*balance*]
2. Do you consider the nurses to spend the necessary time you need with you? [*dedication*]
   1. In case of not: **Reasons**.
3. Do the nurses attend to any extra tasks you ask them in case of need?
   1. In case of not: **Reasons**.

***Preferences expression and shared decision-making situations***

1. Have you chosen something or decided something regarding your care?
   1. [Yes] In which situations?
      1. Have you been able to do that the way you wanted to? [*coincidence*]
   2. [No] Why not? **Reasons**.

***Active participation situations***

1. What is for you “to engage in your care”?
2. Do you think you engage as a patient in the nursing care services you receive?
3. [Yes] In which situations?
   1. Did you want to engage then? And in that way? [*coincidence*]
4. [No] Why not? **Reasons**.

***Professional behaviours***

1. When you have any preference or think about something related to your health condition: how is it to tell the nurses?
   1. [If easy] How do you do it?
   2. [If difficult] Why? **Reasons**.
2. Do you think the nurses listen to you if you want to share something with them?
   1. [Yes] Do the nurses follow your decision to be made?
   2. [No] **Reasons**.
3. Do the nurses do everything for you or encourage you to do something regarding your care? [*taking advantage of capabilities*]

**Additional file 3b.** Interview guide for primary care nurses.

***Preliminary questions***

1. How has your experience with nursing home care been?
2. Do you think you somehow treat older patients differently?
   1. [Yes] In which terms?
   2. [No] Why not? **Reasons**.

***Appointments***

1. When arranging an appointment, do you consider the older patient to establish the time slot for visits? [*care organisation*]

***Nursing care services***

1. Do you think the nursing care services you give are those the older patients really need? [*convenience*]
   1. [Yes] Do you think you give those nursing care services when they need them? [*timely*]
   2. [No] Why not? **Reasons**.
2. Have you allowed the older person to give their opinion regarding the nursing care services you provide or the treatment they receive?
   1. [Yes] In which situations?
   2. [No] Why not? **Reasons**.

***Nursing home visits***

1. Do nursing home visits adapt to older patients´ daily life/lifestyles? [*balance*]
2. Do you consider you spend the necessary time older patients need with them? [*dedication*]
   1. In case of not: **Reasons**.
3. Do you attend to any extra tasks older patients ask you in case of need?
   1. In case of not: **Reasons**.

***Preferences expression and shared decision-making situations***

1. Have you allowed older patients to choose or decide about their care?
   1. [Yes] In which situations?
      1. Have they been able to do that the way they wanted to? [*coincidence*]
   2. [No] Why not? **Reasons**.

***Active participation situations***

1. What is for you “to engage in care”?
2. Do you think older patients engage in your nursing care services?
3. [Yes] In which situations?
   1. Do you think they wanted to engage then? And in that way? [*coincidence*]
4. [No] Why not? **Reasons**.

***Professional behaviours***

1. How do you think it is for older patients to tell you something?
   1. [If easy] How do they do it?
   2. [If difficult] Why? **Reasons**.
2. Do you think you listen to older patients when they want to share something with you?
   1. [Yes] Do you follow their decision to be made?
   2. [No] **Reasons**.
3. Do you do everything for the older patient or encourage them to do something regarding their care? [*taking advantage of capabilities*]
