# Supplementary material for: Active listening, shared decision-making and participation in care among older women and primary care nurses: a critical discourse analysis approach from a gender perspective
Source: BMC Nurs. 2024 Jun 17;23:401. doi: 10.1186/s12912-024-02086-6 (PMC11181639; doi:10.1186/s12912-024-02086-6)
Supplement: Supplementary file 4 — Supplementary Material 4. [file 12912_2024_2086_MOESM4_ESM.docx]

Additional file 4. Magnitude of the derived qualitative findings.

|  | Major themes (magnitude in %) | Minor themes (magnitude in %) | Speech acts (magnitude in %) | Pattern A Major themes (magnitude in %) | Pattern A Minor themes (magnitude in %) | Pattern A Speech acts (magnitude in %) | Pattern B Major themes (magnitude in %) | Pattern B Minor themes (magnitude in %) | Pattern B Speech acts (magnitude in %) | Pattern C Major themes (magnitude in %) | Pattern C Minor themes (magnitude in %) | Pattern C Speech acts (magnitude in %) | Pattern D Major themes (magnitude in %) | Pattern D Minor themes (magnitude in %) | Pattern D Speech acts (magnitude in %) |
| --- | --- | --- | --- | --- | --- | --- | --- | --- | --- | --- | --- | --- | --- | --- | --- |
| Older  women | 3 (30%) | 7 (50%) | 34 (53%) | 0 (0%) | 0 (0%) | 0 (0%) | 1 (25%) | 4 (57%) | 14 (52%) | 1 (50%) | 2 (67%) | 17 (81%) | 1 (50%) | 1 (50%) | 3 (60%) |
| Primary  care nurses | 3 (30%) | 7 (50%) | 30 (47%) | 2 (100%) | 2 (100%) | 11 (100%) | 0 (0%) | 3 (43%) | 13 (48%) | 0 | 1 (33%) | 4 (19%) | 1 (50%) | 1 (50%) | 2 (40%) |
| In common | 4 (40%) | 0 (0%) | 0 (0%) | 0 (0%) | 0 (0%) | 0 (0%) | 3 (75%) | 0 (0%) | 0 (0%) | 1 (50%) | 0 (0%) | 0 (0%) | 0 (0%) | 0 (0%) | 0 (0%) |
| In total | 10 (100%) | 14 (100%) | 64 (100%) | 2 (100%) | 2 (100%) | 11 (100%) | 4 (100%) | 7 (100%) | 27 (100%) | 2 (100%) | 3 (100%) | 21 (100%) | 2 (100%) | 2 (100%) | 5 (100%) |
